# Formulation and *in vitro* evaluation of an anti-inflammatory herbal suppository containing *Peperomia pellucida*

**DOI:** 10.3389/fphar.2026.1718685

**Published:** 2026-02-18

**Authors:** W. A. D. J. Thanishka, D. M. W. L. Dassanayake, D. M. S. R. Dimbulgahamada, W. D. U. S. Fernando, N. T. B. Dias, H. H. V. K. N. De Silva, B. L. C. Samanmali

**Affiliations:** 1 Department of Pharmacy and Pharmaceutical Sciences, Faculty of Health Sciences, CINEC Campus, Malabe, Sri Lanka; 2 Department of Pharmacy, Faculty of Allied Health Sciences, University of Peradeniya, Peradeniya, Sri Lanka; 3 Department of Pharmacy, Faculty of Allied Health Sciences, General Sir John Kotelawala Defence University, Ratmalana, Sri Lanka

**Keywords:** 5-lipoxygenase inhibition, hemolysis inhibition, herbal suppository, Peperomia pellucida, rectal drug delivery systems

## Abstract

**Introduction:**

Adverse effects associated with the anti-inflammatory medicines highlight the need for safer alternatives. The present study aimed to evaluate the anti-inflammatory characteristics of *Peperomia pellucida* and to develop an anti-inflammatory herbal suppository using the plant extract.

**Methods:**

Methanolic and aqueous extracts of *P. pellucida* were assessed using 5-lipoxygenase inhibition assay, human red blood cell (HRBC) membrane stabilization assay, and egg albumin denaturation assay. The aqueous extract was characterized by gas chromatography-mass spectrometry (GC-MS) and Fourier transform infrared (FTIR) analysis. Test suppositories containing the aqueous extract of *P. pellucida* were formulated using several bases, physically characterized, and subjected to stability assessment for 60 days. Triplicate results were statistically analyzed at p < 0.05.

**Results:**

The highest percentage inhibition of hemolysis was exhibited by the aqueous extract at 5000 μg/mL (88.42%) compared to the methanolic extract (58.08%) at the same concentration in the HRBC membrane stabilization assay. This observation was further supported by the lower IC_50_ value of the aqueous extract (861.1 μg/mL). GC-MS analysis indicated the presence of a high abundance of long-chain fatty acids in the aqueous extract, including palmitic and myristic acids. The formulated suppositories exhibited acceptable weight uniformity (1.73 ± 0.00 g), hardness (8.12 ± 0.00 N), pH (7.53 ± 0.05), liquefaction time (534.2 ± 3.4 s), melting point (36°C–42 °C), and *ex vivo* drug permeation. No significant differences were observed in the quality parameters between day 0 and day 60 (p > 0.05).

**Discussion:**

The relatively low IC_50_ value of the aqueous extract of *P. pellucida* demonstrated strong membrane stabilization, suggesting that solvent selection significantly influences the efficacy of botanical drugs. Fatty acids identified by GC-MS may account for the observed biological membranes stabilizing activity. The successful formulation of the aqueous extract into a suppository was evidenced by its customary physicochemical properties. The results of *ex vivo* permeation study and the absence of significant changes following the 2-month stability period confirm the efficacy and integrity of the dosage form. These findings support the potential of *P. pellucida* as a source for the development of novel, plant-based rectal therapeutics for inflammation-related conditions.

## 1 Introduction

Inflammation is an intrinsic physiological response of the body to damage of living tissues. As the initial line of defense against harmful molecules and external invaders, the innate immune system triggers acute inflammatory responses. Redness, pain, swelling, and heat are common symptoms associated with inflammation (Nguyen et al., 2023). Anti-inflammatory drugs (e.g., corticosteroids and nonsteroidal anti-inflammatory drugs) are widely used to reduce or control inflammation during severe responses, however, they are associated with various adverse effects. Novel biologics containing purified antibodies (e.g., infliximab and adalimumab) target specific cytokines that mediate inflammatory responses (Placha and Jampilek, 2021; Amponsah et al., 2022).

Botanical drugs have emerged as a major focus of contemporary pharmaceutical research, primarily due to their rich biodiversity and presence of structurally diverse metabolites. Crude plant extracts are an imperative component in these investigations, as they contain complex mixtures of metabolites that often act synergistically to produce enhanced therapeutic effects compared to isolated drugs (Donkor et al., 2023). Such synergism may result in broader pharmacological actions and improved bioavailability. The multifaceted pharmacological profiles of crude plant extracts make them promising candidates for managing conditions such as cancer, microbial resistance, and chronic inflammatory disorders, where multi-component interventions may be more effective than single-molecule drugs (Sychrová et al., 2020; Humne et al., 2023). Advanced analytical techniques, such as gas chromatography-mass spectrometry (GC-MS), facilitate the identification of complex chemical compositions in crude plant extracts. Nevertheless, extensive scientific investigations are required to confirm the safety and efficacy of botanical drugs (Ribeiro et al., 2018). *Peperomia pellucida* (L.) Kunth is a medicinal plant belonging to the family Piperaceae and is commonly found in moist, shaded areas of tropical regions. Traditionally, *P. pellucida* has been used across various ethnicities to treat a wide range of ailments, including inflammatory conditions such as eczema, boils, and skin wounds, as well as respiratory disorders, epilepsy, hypertension, and kidney diseases (Amarathunga and Kankanamge, 2017). This plant drug represents a promising therapeutic candidate for inflammatory diseases, supported by its antipyretic, analgesic, and antioxidant activities reported in previous *in silico*, *in vitro*, and *in vivo* studies (Ahmad et al., 2023).

Suppository dosage forms are designed for insertion into body orifices, where they dissolve, melt, or soften to exert localized or systemic effects. They provide several advantages, including ease of administration for patients experiencing nausea or vomiting and suitability when oral administration is not feasible (Rao et al., 2023). In recent years, suppositories containing anti-inflammatory, analgesic, antipyretic, sedative, and hypnotic agents have been widely marketed (Purohit et al., 2018). Accordingly, plant-based therapies formulated as suppositories may provide discreet and effective treatment for acute inflammatory conditions, particularly those localized to the lower gastrointestinal tract. Such formulations allow targeted and sustained delivery of bioactive metabolites, aligning with the growing demand for natural medicines (Saleem et al., 2024). Although the anti-inflammatory properties of *P. pellucida* have been previously documented (Fakayode et al., 2021), its development into a standardized rectal dosage form with potential medicinal benefits for localized therapy has not been explored. This study integrates multiple *in vitro* bioassays, phytochemical characterization, and the development of a rectal dosage form incorporating *P. pellucida* extract. To the best of our knowledge, this is the first report describing the formulation and stability evaluation of a suppository based on *P. pellucida*. By standardizing this formulation and systematically evaluating its physicochemical properties, the present study extends the therapeutic application of the test plant beyond conventional preparations.

## 2 Materials and methodology

### 2.1 Ethical clearance

Ethical approval for this study was obtained from the Ethics Review Committee of CINEC Campus, Sri Lanka, which involved the collection of human whole blood samples (Approval certificate no. ERC/CINEC/2024/004).

### 2.2 Plant collection and authentication

Fresh *P. pellucida* plant material was collected from the Western Province of Sri Lanka (6.7156° N, 79.9391° E) in February 2024. The collected material was prepared into a herbarium specimen and authenticated by the Bandaranaike Memorial Ayurvedic Research Institute, Maharagama, Sri Lanka (Acc. No. 3175).

### 2.3 Preparation of *Peperomia pellucida* plant extracts

Clean, fresh aerial parts of *P. pellucida* were shade-dried until a constant weight was achieved. Subsequently, 30 g of the dried plant material was refluxed with 100 mL of methanol and 100 mL of distilled water separately at 70 °C for 2 hours. The extracts were filtered by suction filtration and dried by solvent evaporation. The percentage yields of extraction were 4.22% and 5.57% for methanolic and aqueous extracts, respectively.

### 2.4 Determination of *in vitro* anti-inflammatory activity

#### 2.4.1 5-Lipoxygenase inhibition assay

The assay was performed using 5-lipoxygenase (5-LOX) enzyme derived from soybeans. Both dried plant extracts were dissolved in distilled water to prepare concentrated stock solutions at 100 μg/mL. A reaction mixture containing 260 µL of 0.1 M phosphate buffer (pH 7.0), 100 µL of 5-LOX solution, and 30 µL of plant extract was pre-incubated at 37 °C for 5 min. The reaction was initiated by adding 10 µL of freshly prepared 5 mM linoleic acid in ethanol and incubated for an additional 3 min. The percentage inhibition of enzyme activity was calculated by measuring the UV-visible absorbance of the reaction product at 234 nm. Zileuton was used as positive control.

#### 2.4.2 Human red blood cell (HRBC) membrane stabilization assay

Fresh whole blood was collected from healthy adult volunteers under aseptic conditions following informed written consent. Three milliliters of blood was immediately mixed with an equal volume of Alsever’s solution. The mixture was centrifuged at 3000 rpm for 10 min, the supernatant was discarded, and the cells were washed with isotonic saline. Methanolic and aqueous extract powders were dissolved in distilled water to prepare a concentration series ranging from 5000 to 125 μg/mL. A volume of 600 µL of each extract was separately mixed with 1 mL of 0.15 M phosphate buffer (pH 7.4), 2 mL of 0.36% hyposaline, and 0.4 mL of a 10% v/v HRBC suspension. The mixtures were incubated at 37 °C for 30 min and then centrifuged at 3000 rpm for 10 min. The absorbance of the supernatant was measured at 560 nm. Diclofenac sodium (250–7.81 μg/mL) was used as the standard drug, while phosphate buffer served as the control. The percentage inhibition of hypotonicity-induced hemolysis was calculated according to a previously described method (Javed et al., 2020).

#### 2.4.3 Egg protein denaturation assay

Egg albumen (5 mL) was mixed with 10 mL of distilled water, gently swirled without inducing denaturation, and filtered through a clean muslin cloth. Methanolic and aqueous extract powders were dissolved in distilled water to prepare a concentration series ranging from 1000 to 62.5 μg/mL. The reaction mixture consisted of 2.8 mL of freshly prepared phosphate-buffered saline (pH 6.3), 2 mL of plant extract, and 0.2 mL of diluted egg albumen. These mixtures were heated in a water bath at 37 °C for 15 min, followed by gradual heating to 70 °C, at which temperature the samples were maintained for 5 min. The samples were then cooled to 28 °C, and absorbance was measured at 660 nm. Diclofenac and phosphate buffer were used as positive and negative controls, respectively. The percentage inhibition of protein denaturation was calculated using a reported method (Heendeniya et al., 2018).

### 2.5 Fourier transform infrared (FTIR) analysis of the extract

Functional groups present in the aqueous extract of *P. pellucida* were analyzed using FTIR spectroscopy equipped with an attenuated total reflectance (ATR) accessory (PerkinElmer UATR II, United States). A small amount of the extract was placed directly onto the diamond ATR crystal. Spectra were recorded over the wavenumber range of 4000–400 cm^-1^ at a resolution of 4 cm^-1^, with 32 scans averaged per spectrum.

### 2.6 Analysis of phytochemical profile using GC-MS

GC-MS analysis of the aqueous extract of *P. pellucida* was performed using an Agilent Technologies 7890B GC system coupled with a 5977A mass selective detector, a 7890B autosampler, and GC-MS ChemStation software with compound identification based on NIST14 and W9N11 libraries. For sample preparation, 0.2 g of the dried extract was dissolved in 10 mL of ethanol, and 1 µL of the filtrate was injected into the GC-MS system. Separation was achieved using an Agilent HP-5 ms Ultra Inert capillary column (30 m × 250 μm × 0.25 µm), with helium as the carrier gas at a constant flow rate of 1.0 mL/min. The GC oven temperature was initially set at 50 °C and held for 2 min, followed by a linear temperature increase at a ramp rate of 7 °C/min until the final temperature of 300 °C reached. The injector temperature was maintained at 250 °C.

### 2.7 Fabrication and calibration of suppository mold

#### 2.7.1 3D printing of the suppository mold

Siemens NX 12.0 software was used to design and 3D-print a suppository mold using polylactic acid plus (PLA+) plastic (melting point: 170 °C–180 °C). The molds were torpedo-shaped, with a length of 22.56 mm and varying diameters (upper: 7.00 mm; middle: 8.40 mm; lower tip: 4.32 mm). Each mold consisted of five separate compartments.

#### 2.7.2 Calibration of molds

A gelatin mixture was prepared by dissolving gelatin in hot distilled water with gentle stirring. The warm mass was poured into dry suppository mold cavities and allowed to set. The molded suppositories were carefully removed, and the total weight of suppositories from the entire mold was recorded. The average weight per cavity was then calculated.

### 2.8 Preparation of placebo suppositories

The excipients used for the preparation of placebo suppositories were selected based on published literature (Freisz et al., 2024). The proportions of each ingredient were optimized using a trial-and-error approach to achieve suitable suppository hardness. Polyethylene glycol (PEG) 4000 was selected as a primary base due to its hydrophilic nature and ability to produce a hard mold, while glycerin was incorporated as a plasticizer to adjust suppository hardness. For the preparation of aqueous-base placebo suppositories, PEG 4000, glycerin, gelatin, and distilled water were combined in different ratios, designated as A1 - A10 (Table 1). Cocoa butter (100%) was melted and poured into the suppository mold (Composition O1) to prepare oleaginous-base placebo suppositories. Various combinations of PEG 4000, cocoa butter, glycerin, and distilled water were used to formulate miscellaneous-base placebo suppositories, which were indicated as M1 - M6 (Table 2). All bases were warmed separately, poured into the molds, and allowed to solidify at room temperature.

**TABLE 1:** Composition of tested aqueous base suppository formulations.

Ingredients	A1	A2	A3	A4	A5	A6	A7	A8	A9	A10
Gelatine (g)	1.00	1.00	1.50	0.90	0.85	0.75	0.50	0.75	0.75	0.00
Glycerin (g)	7.00	6.00	6.00	6.00	6.00	6.00	6.50	2.00	1.00	0.87
PEG 4000 (g)	0.00	1.00	0.50	1.10	1.15	1.25	1.00	5.25	6.25	6.73
Distilled water (g)	2.00	2.00	2.00	2.00	2.00	2.00	2.00	2.00	2.00	2.40
Total (g)	10.00	10.00	10.00	10.00	10.00	10.00	10.00	10.00	10.00	10.00

**TABLE 2:** Composition of miscellaneous base suppository formulations.

Ingredients	M1	M2	M3	M4	M5	M6
PEG 4000 (g)	2.00	2.50	2.70	3.00	3.25	3.35
Cocoa butter (g)	5.00	4.50	4.30	4.15	3.80	3.75
Glycerin (g)	1.00	1.00	1.00	0.75	0.75	0.50
Distilled water (g)	2.00	2.00	2.00	2.10	2.20	2.40
Total (g)	10.00	10.00	10.00	10.00	10.00	10.00

### 2.9 Formulation of *Peperomia pellucida*-loaded suppositories

The strength of *P. pellucida* in the test suppositories was determined by comparing the half-maximal inhibitory concentration (IC_50_) of plant extracts in the HRBC membrane stabilization assay with that of diclofenac sodium. Based on favorable physical hardness character, compositions A10, O1, and M6 were selected from each base category for the formulation of test suppositories. Once the plant extract (480.0 mg per suppository unit) was completely dissolved in distilled water, the solution was gradually incorporated into the molten bases of A10, M6, and O1 separately. Each mixture was stirred continuously for 5 min until homogeneity was achieved, poured into the suppository molds, and allowed to cool. Finally, benzyl alcohol was applied as a preservative coating.

### 2.10 Characterization and quality control of suppositories

The formulated herbal suppositories and placebo formulations were initially evaluated for physical appearance by visual inspection. Only suppository batches with acceptable appearance (which was Composition A10) were subjected to further quality control tests, according to the methods described below (Aremu et al., 2019).

#### 2.10.1 *In vitro* bioactivity

Ten placebo suppositories and ten plant extract-incorporated suppositories were weighed separately and dissolved in phosphate buffer (pH 7.4) to obtain a final concentration of 20 mg/mL. The HRBC membrane stabilization assay was then performed for each sample as described previously.

#### 2.10.2 Appearance

Ten suppositories were randomly selected, and their color and shape were evaluated.

#### 2.10.3 Weight uniformity

Ten suppositories were randomly selected and weighed individually using an analytical balance. The mean weight and standard deviation were calculated.

#### 2.10.4 Melting point

Ten randomly selected suppositories were placed in separate beakers, and thermometers were inserted. Each beaker was immersed to a depth of 6 cm in a water bath with a controlled temperature increase of 1 °C every 2 min. The melting point was recorded as the temperature at which the suppository first began to melt.

#### 2.10.5 Liquefaction time and pH

The phosphate buffer (60 mL, pH 7.4) was heated in a shaking water bath to 37 °C ± 1 °C. Liquefaction time was determined by immersing ten suppositories individually in the buffer and recording the time required for complete melting. The pH of each melted suppository was measured using a portable pH meter.

#### 2.10.6 Hardness

The crushing strength of the suppositories was determined using a Monsanto-type manual hardness tester. Ten suppositories were randomly selected, and the force at which each suppository fractured was recorded.

#### 2.10.7 Determination of *in vitro* drug release over time

Phosphate buffer (pH 7.4) was prepared and transferred to a 600 mL beaker. To simulate *in vivo* conditions, the temperature was maintained at 37 °C, and the magnetic stirrer was set to a moderate rotation speed. A suppository was carefully placed in the buffer to initiate dissolution. At 30 s intervals, 10 mL samples were withdrawn and subjected to the HRBC membrane stabilization assay using the previously described method. The release profile was determined based on the bioactivity of the collected samples.

### 2.11 Determination of *ex vivo* permeation of the extract

Eggshell membranes were prepared by dissolving fresh chicken eggshells in 3 M hydrochloric acid, followed by thorough rinsing with phosphate buffer. The suppository was placed on the donor side of the permeation apparatus, which was immersed in phosphate buffer (pH 6.9) containing 20% ethanol as the receiver medium. The system was maintained at 37 °C ± 0.5 °C with continuous stirring at 40 rpm. Samples were withdrawn from the receiver compartment at predetermined time intervals, and fresh preheated buffer was added to maintain sink conditions. The collected samples were analyzed using the previously described method to determine hemolysis inhibition corresponding to each time point.

### 2.12 Preliminary stability studies

The prepared aqueous plant extract-incorporated aqueous base suppositories were stored at 4 °C in a refrigerator (Hanning et al., 2021). After 2 months, the suppositories were re-evaluated for physical stability by conducting the quality control tests described previously.

### 2.13 Statistical analysis

All experiments were performed in triplicate (n = 3), and results were expressed as mean ± standard deviation (SD). Statistical analysis was conducted using the paired t-test at significance level of 0.05. GraphPad Prism (version 10) was used to generate graphs and to calculate median inhibitory values by regression analysis.

## 3 Results

### 3.1 *In vitro* anti-inflammatory activity of *Peperomia pellucida*

#### 3.1.1 5-Lipoxygenase inhibition

At the tested concentration of 100 μg/mL, the aqueous and methanolic plant extracts demonstrated moderate enzyme inhibitory effects, with inhibition values of 58.22% ± 0.20% and 68.01% ± 0.15%, respectively. The standard drug zileuton showed 88.74% ± 0.22% inhibition.

#### 3.1.2 Human red blood cell membrane stabilization

**FIGURE 1:**
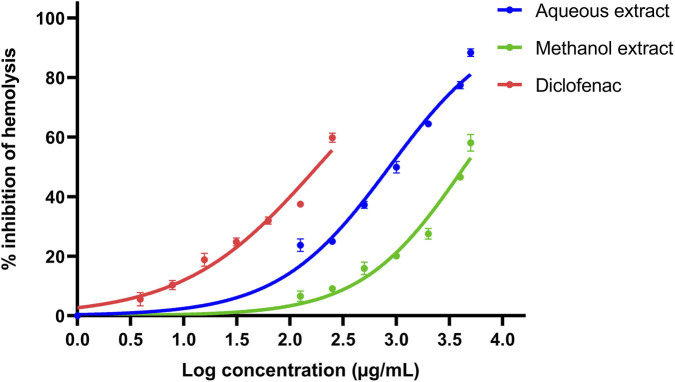
Inhibitory activity of hypotonicity-induced hemolysis by *Peperomia pellucida* extracts and diclofenac sodium standard.

At the concentration of 5000 μg/mL, the aqueous extract exhibited the highest inhibition of *in vitro* hemolysis (88.42% ± 1.31%), whereas the lowest activity (23.72% ± 2.09%) was observed at 125 μg/mL. The methanolic extract also showed dose-dependent activity, where it inhibited hemolysis by 58.08% ± 2.81% at the highest concentration (5000 μg/mL), with decreasing activity at lower concentrations. Diclofenac sodium inhibited *in vitro* hemolysis by 59.81% ± 0.53% at the concentration of 250 μg/mL. The aqueous extract exhibited a lower IC_50_ value (861.1 μg/mL) than the methanolic extract (4364.0 μg/mL), indicating comparatively stronger anti-inflammatory activity. Diclofenac sodium demonstrated the lowest IC_50_ value (179.8 μg/mL), confirming its potent activity (Figure 1).

#### 3.1.3 Egg protein denaturation inhibition

**FIGURE 2:**
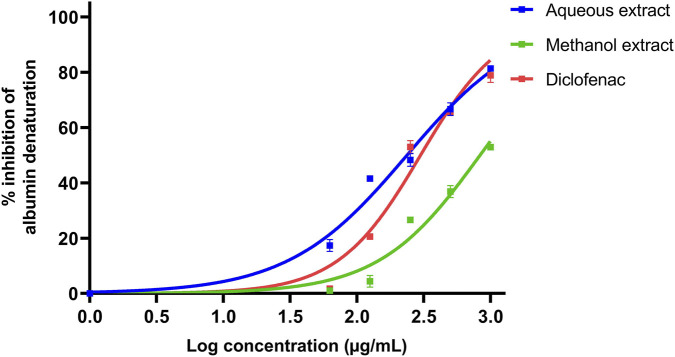
Inhibitory activity of heat-induced protein denaturation by *Peperomia pellucida* extracts and diclofenac sodium standard.

At the highest test concentration (1000 μg/mL), the aqueous extract showed the greatest inhibition of protein denaturation (81.38% ± 0.16%), while inhibition decreased to 17.36% ± 2.16% at the lowest concentration tested (62.5 μg/mL). The methanolic extract of *P. pellucida* demonstrated 52.92% ± 0.10% inhibition at 1000 μg/mL, which decreased to 1.03% ± 0.17% at 62.5 μg/mL. At 1000 μg/mL, diclofenac sodium exhibited 78.93% ± 2.56% inhibition. Notably, the aqueous extract showed higher inhibition of protein denaturation than diclofenac at lower concentrations. The aqueous extract exhibited an IC_50_ value of 236.0 μg/mL, while the methanolic extract showed a higher IC_50_ value of 832.4 μg/mL indicating lower activity. Diclofenac sodium showed an IC_50_ value of 298.7 μg/mL (Figure 2).

### 3.2 FTIR analysis of aqueous extract of *Peperomia pellucida*

**FIGURE 3:**
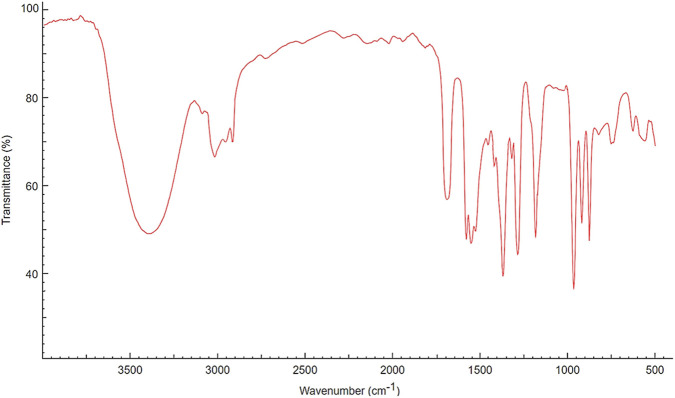
FTIR spectrum of the aqueous extract of *Peperomia pellucida*.

The FTIR spectrum of the aqueous extract of *P. pellucida* (Figure 3) revealed a broad absorption band in the region of 3400–3300 cm^-1^, indicative of hydroxyl-containing metabolites such as phenolics, alcohols, and carbohydrates. Prominent absorption bands observed at approximately 2920–2850 cm^-1^ wavenumbers are attributed to aliphatic C–H stretching, suggesting the presence of long-chain fatty acids. A distinct peak in the range of 1730–1700 cm^-1^ corresponded to C=O stretching vibrations associated with carbonyl groups of fatty acids or ester functionalities. Peaks in the region of 1250–1050 cm^-1^ were assigned to C–O stretching vibrations of alcohols and polysaccharides, while bands below 1000 cm^-1^ indicated aromatic bending modes.

### 3.3 GC-MS profile of aqueous extract of *Peperomia pellucida*

**FIGURE 4:**
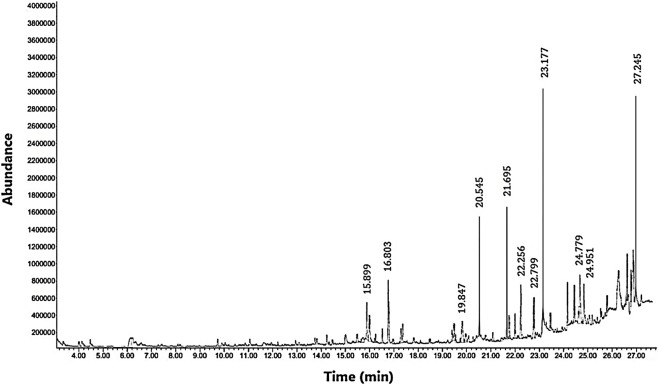
Gas chromatography-mass spectrometry (GC-MS) profile of crude aqueous extract of *Peperomia pellucida*.

The GC-MS analysis of the aqueous crude extract of *P. pellucida* revealed a diverse phytochemical composition. The chromatogram (Figure 4) indicated that the most abundant metabolites were long-chain fatty acids, including n-hexadecanoic acid (palmitic acid), hexadecanoic acid derivatives, and tetradecanoic acid (myristic acid). The identified phytochemicals with ≥1.0% relative abundance are presented in Table 3. Chemical structures were adapted from the National Institute of Standards and Technology database using the Chemical Abstracts Service (CAS) numbers provided by the GC-MS library (National Institute of Standards and Technology, 2025).

**TABLE 3:** Most abundant phytochemicals in *Peperomia pellucida* aqueous extract (GC-MS).

Rank	Area %	Compound name	Retention time (min)	Chemical structure^a^
1	18.09	n-hexadecanoic acid	23.177	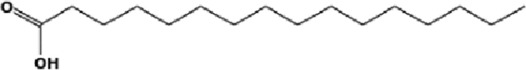
2	10.20	Hexadecanoic acid, 2-hydroxy-1-(hydroxymethyl)ethyl ester	27.245	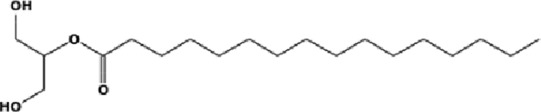
3	9.98	Tetradecanoic acid	20.545	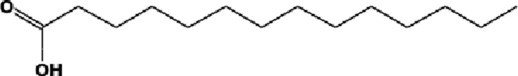
4	7.27	2-pentadecanone, 6,10,14-trimethyl	21.695	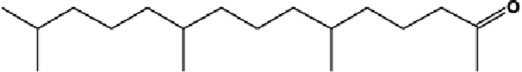
5	4.26	9-octadecenoic acid (E)-	24.779	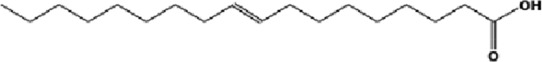
6	3.79	Benzoic acid, 4-ethoxy-, ethyl ester	16.803	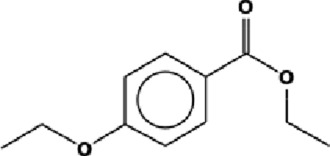
7	3.48	Neophytadiene	22.256	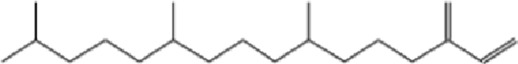
8	2.28	1-(4-ethoxyphenyl)propan-1-ol	15.899	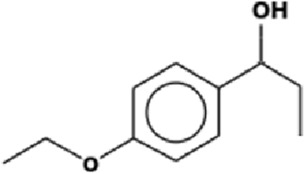
9	2.17	7,9-di-tert-butyl-1-oxaspiro(4,5)deca-6,9-diene-2,8-dione	22.799	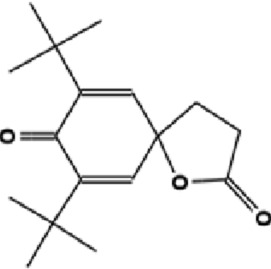
10	2.13	2H-pyran-2-one, tetrahydro-4-hydroxy-6-pentyl-	19.847	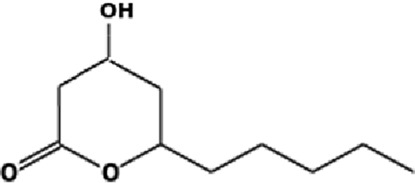
11	1.95	Octadecanoic acid	24.951	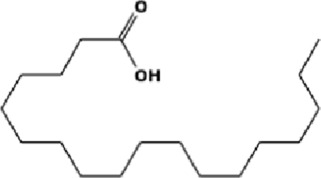
12	1.82	2H-pyran-2-one, 5,6-dihydro-6-pentyl-	16.013	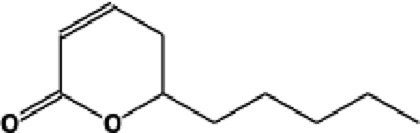
13	1.72	1-octadecene	24.316	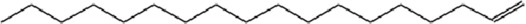
14	1.54	Glycerin	6.182	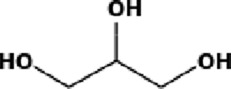
15	1.45	Phytol	24.579	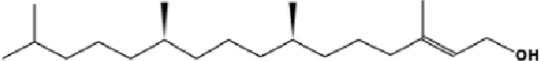
16	1.33	9,12-octadecadienoic acid (Z,Z)-	24.739	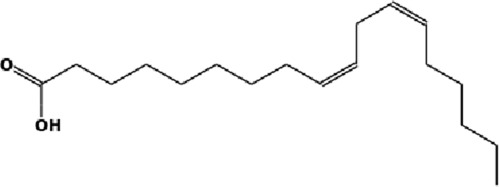
17	1.29	Benzeneethanol, 4-hydroxy-	15.034	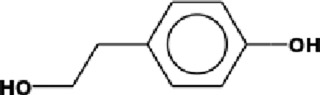
18	1.28	Neophytadiene	22.027	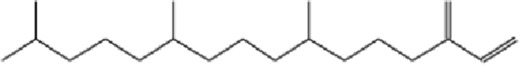
19	1.21	Phenol, 2,2′-methylenebis[6-(1,1-dimethylethyl)-4-methyl-	26.793	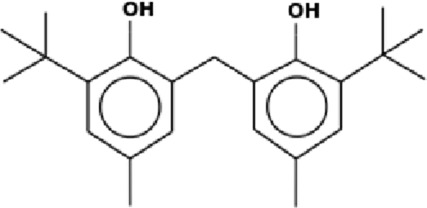
20	1.17	2-Pentadecanone, 6,10,14-trimethyl	21.792	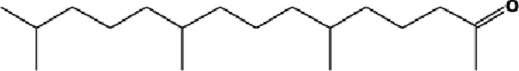
21	1.15	Vitamin E	27.051	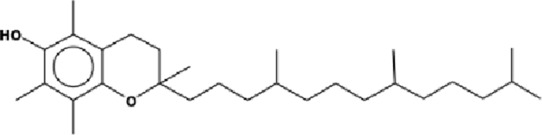
22	1.06	1,2-Dimethoxy-4-(2-methoxyethenyl)benzene	17.403	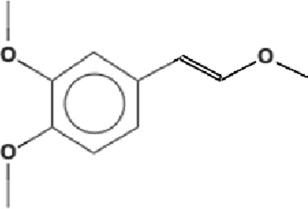

^a^ Chemical structures were adapted from the National Institute of Standards and Technology (2025) by using the Chemical Abstracts Service (CAS) number provided by the GCMS library.

### 3.4 Calibration of mold and suppository formulation

The capacity of the suppository mold was determined to be 1.71 ± 0.00 g. Many of the aqueous-base placebo suppositories (A1 - A9) and miscellaneous-base placebo suppositories (M1 - M5) were physically unstable and/or failed to solidify adequately. Consequently, A10, O1, and M6 were identified as suitable compositions for suppository formulation. While the A10 and M6 suppositories remained stable following incorporation of the plant extract, the O1 formulation exhibited poor mixing and was therefore excluded from further development. The M6 suppository showed poor dispersion and phase separation of the plant extract, whereas the A10 suppository demonstrated uniform dispersion of the active ingredient. Accordingly, the A10 formulation was selected as the optimal suppository for further evaluation.

### 3.5 Quality control of the formulation

The physicochemical properties of the *P. pellucida* aqueous extract-incorporated herbal suppository were evaluated and compared with those of the A10 placebo suppository. All assessed quality control parameters of the test formulation were within prescribed limits (Table 4). Reference values were adapted from previous studies (Sahoo et al., 2017). The plant drug-incorporated suppositories inhibited hemolysis by 52.10% ± 0.15% in the HRBC membrane stabilization assay. This was statistically significant (p < 0.05) compared with the placebo suppository, which inhibited only 5.88% ± 0.15% hemolysis.

**TABLE 4:** Physicochemical properties of *Peperomia pellucida* suppository.

Parameters	Herbal suppository	Suppository placebo	Reference value^a^
Shape	Torpedo	Torpedo	Torpedo shape
Color	Brownish color	White color	Not applicable
Weight uniformity (g)	1.73 ± 0.00	1.20 ± 0.00	Weight difference not to be differ from average weight by more than 10%
Melting point (°C)	36–42	36–41	35–38
Liquefaction time (s)	534.2 ± 3.4	300.8 ± 0.1	<30 min
Hardness (N)	8.12 ± 0.00	8.00 ± 0.00	<20
pH	7.53 ± 0.05	7.21 ± 0.04	7.0–8.0

^a^ Reference values were adapted from literature.

Data values are given as mean ± standard deviation (n = 10).

### 3.6 *Ex vivo* permeation of the plant drug

Table 5 presents the *ex vivo* permeation data of the *P. pellucida*-loaded suppository across the eggshell membrane. A progressive increase in biological activity was observed during the initial phase of the experiment, indicating gradual permeation of bioactive metabolites through the membrane. HRBC membrane stabilization activity increased from 10.58% at 0.5 min to 38.55% at 10 min. Maximum activity was recorded at 15 min (41.52%), representing the time point of optimal permeation and biological effectiveness. Thereafter, a gradual decline in activity was observed.

**TABLE 5:** *Ex vivo* permeation data of the *Peperomia pellucida*-loaded suppository.

Time (min)	Permeation of the active species (% inhibition of hemolysis)
0.5	10.58 ± 2.01
1	16.25 ± 1.83
2	28.05 ± 2.05
5	33.40 ± 2.11
10	38.55 ± 1.52
15	41.52 ± 1.55
20	36.33 ± 1.77
25	33.00 ± 1.86
30	32.52 ± 2.10

### 3.7 Stability assessment of the formulation

Physical appearance, weight uniformity, melting point, and pH remained consistent between the day of manufacture (day 0) and after 2 months of storage (day 60), indicating satisfactory physical stability (Table 6). Liquefaction time and hardness increased slightly during the storage period, however, these changes were statistically comparable (p > 0.05).

**TABLE 6:** Stability tests for *Peperomia pellucida* suppository.

Parameters	Test suppository on day 0	A10 suppository on day 60	Variation
Shape	Torpedo	Torpedo	No difference
Color	Brownish color	Brownish color	No difference
Melting point (°C)	36–42	36–42	No difference
Weight uniformity (g)^a^	1.73 ± 0.00	1.73 ± 0.00	p > 0.005
Liquefaction time (s)^a^	534.2 ± 3.4	542.0 ± 2.1	p > 0.005
Hardness (N)^a^	8.12 ± 0.00	8.17 ± 0.20	p > 0.005
pH^a^	7.53 ± 0.05	7.51 ± 0.04	p > 0.005

^a^ Values are given as mean ± standard deviation (n = 10), statistical analysis by paired samples t-test at 0.05 significance level.

**FIGURE 5:**
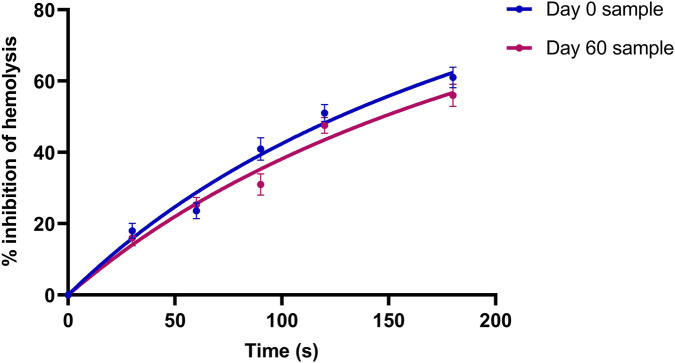
Release profile of the test herbal suppository on day 0 and day 60 (by HRBC assay).

In the *in vitro* drug release study of the aqueous extract-incorporated suppository, HRBC membrane stabilization (inhibition of hemolysis) gradually increased to 61.22% ± 1.24% at 180 s on day 0. After 2 months of storage, the release profile remained comparable, with a maximum inhibition of 56.41% ± 1.16% at 180 s (Figure 5). The variation in biological activity was not statistically significant (p > 0.05).

## 4 Discussion

The results of the 5-lipoxygenase inhibition assay indicate that *P. pellucida* contains constituents capable of modulating enzymatic pathways involved in inflammation, albeit with lower potency than the synthetic drug. This finding supports the hypothesis that moderate therapeutic effects of medicinal plant extracts underscore their potential as adjunctive therapies (Usatiuc et al., 2024). The aqueous extract of *P. pellucida* demonstrated significant dose-dependent HRBC membrane stabilization, with >88% inhibition at 5000 μg/mL. Conversely, the methanol extract showed lower inhibition at the same concentration. The aqueous extract performed better in the present study, contrary to other studies reporting superior membrane-stabilizing activity of methanolic plant extracts (Khan et al., 2022). Lysosomal membrane disruption during inflammation results in the release of enzyme components that cause a variety of pathological conditions. As human red blood cell membranes are biochemically similar to lysosomal membranes in their phospholipid bilayer structure, inhibition of hypotonicity-induced hemolysis of red blood cells can be used to mimic anti-inflammatory mechanisms associated with lysosomal membrane stabilization (Akbor et al., 2024). Hypotonic systems cause red blood cells to rupture due to excessive fluid accumulation within their membranes. If a test plant extract possesses considerable anti-inflammatory activity, this hemolytic process will be inhibited, thereby reflecting the therapeutic potential of the test material (Amaro et al., 2021). Mechanistically, the parallel effects observed in membrane stabilization and enzyme inhibition assays suggest a plausible pharmacodynamic model, in which membrane stabilization prevents upstream release of arachidonic acid from lysosomes, while 5-lipoxygenase inhibition obstructs downstream leukotriene formation (Gilbert et al., 2021).

In the protein denaturation assay, the aqueous extract exhibited a dose-dependent response with substantial anti-inflammatory activity. Inhibition of protein denaturation exceeded 81% at 1000 μg/mL and decreased to 17.36% at 62.5 μg/mL. In contrast, the methanol extract showed comparatively lower activity, with 52.92% inhibition at 1000 μg/mL. Another study reported that methanolic extracts of *P. pellucida* prepared from both fresh and dried plant samples inhibited albumin denaturation at varying doses (Fakayode et al., 2021). The egg albumin denaturation assay is based on the assertion that anti-inflammatory agents stabilize protein structure and prevent denaturation. Protein denaturation is a common process associated with tissue damage and inflammation and is linked to the expression of a wide range of pro-inflammatory antigens. Therefore, this assay suggests that substances capable of preventing egg albumin denaturation may possess promising anti-inflammatory properties (Yesmin et al., 2020). The observed superior anti-inflammatory activities of aqueous extract of *P. pellucida* may be attributed to the increased presence and potency of hydrophilic metabolites in the extract. Methanolic extraction may yield additional nonpolar constituents that do not contribute to anti-inflammatory activity under the aqueous assay conditions. The higher activity observed in the aqueous extract thus likely reflects differences in extract composition as well as assay compatibility.

The *in vivo* acute and sub-chronic safety of the plant extract has been evidenced at a dose of 500 mg/kg in previous work, without any hematological abnormalities or mortality in test mice. The histopathological evaluation has further reported no significant tissue alterations in liver following administration of the extract, suggesting the absence of toxicity under the tested conditions (Tuan and Men, 2024). In another related acute toxicity study, female Sprague-Dawley rats have shown similar observations at a 5000 mg/kg dose of *P. pellucida* (Zamri et al., 2020). The excessive accumulation of certain metals usually raises toxicological concerns in botanical drugs, however, *P. pellucida* has been shown to contain nutritionally relevant macro-minerals (K, Na, and Ca) with negligible levels of heavy metals such as Se and Pb (Afolabi et al., 2025).

FTIR and GC-MS analyses of the aqueous crude extract of *P. pellucida* revealed a diverse phytochemical profile dominated by fatty acids. The principal metabolites identified were n-hexadecanoic acid (18.09%), hexadecanoic acid 2-hydroxy-1-(hydroxymethyl)ethyl ester (10.20%), tetradecanoic acid (myristic acid, 9.98%), and oleic acid (4.26%). Other bioactives, including neophytadiene, phytol, linoleic acid, octadecanoic acid, and vitamin E, may contribute supplementarily to the observed bioactivity. Minor metabolites such as phenolic derivatives, alcohols, aldehydes, and hydrocarbons may also exert synergistic biological effects (Zhang et al., 2019). Fatty acids are known to stabilize biological membranes by intercalating into lipid bilayers, thereby enhancing structural integrity and preventing hemolysis. Additionally, they may inhibit protein denaturation through hydrophobic interactions with protein structures (Resh, 2013; Samovski et al., 2023). The long chain fatty acids have been reported to exhibit stronger inhibitory activity against cyclooxygenase-1 (COX-1) enzyme while demonstrating comparatively weaker inhibition of other cyclooxygenases. In addition, previous investigations have demonstrated the appreciable inhibitory activity of fatty acids-rich plant extracts against 5-lipoxygenase. Recent *in silico* studies have further supported these findings by indicating binding affinity of fatty acids to the active sites of inflammatory enzymes (Atolani et al., 2019; Maslov et al., 2025; Jaikanth et al., 2025). Collectively, these mechanisms provide a biochemical basis for the anti-inflammatory activities observed in *P. pellucida* and other fatty acid-rich plant extracts.

The suppository mold was designed using 3D printing and manufactured from polylactic acid (PLA), a material that offers high thermal stability even at elevated temperatures. This property minimizes the risk of deformation or compromise of suppository integrity during preparation (Jin et al., 2019). Placebo formulations A1 - A9 exhibited various quality defects, including excessive elasticity, stickiness, and a creamy texture, possibly due to excessive gelatin content or insufficient PEG 4000. These issues adversely affected the physical properties of the suppositories, rendering them unsuitable for further development. Formulation A10, which excluded gelatin and incorporated a higher concentration of PEG 4000, resulted in a hard and stable suppository. A previous study employing a heat-molding technique similar to that used in the present work reported that suppositories containing flax (*Linum usitatissimum*) extracts exhibited acceptable physical quality and favorable therapeutic effects, including laxative activity. That study highlighted potential applications of flax suppositories in the management of hemorrhoids and anorectal bacterial infections (Mishra et al., 2022). Although the eggshell membrane was employed as a simple, reproducible model for preliminary permeation assessment, it does not fully replicate the anatomical or biochemical characteristics of rectal mucosa. Therefore, future *ex vivo* investigations should employ biologically relevant membranes (such as porcine rectal tissue) to improve physiological and translational relevance. Stability analysis of the *P. pellucida* aqueous extract suppository after 2 months demonstrated retention of most physical properties and performance parameters, with only negligible changes. The torpedo-shaped, brownish suppositories retained their appearance throughout the study period. Weight uniformity remained constant at 1.73 g, while melting point and pH did not show significant variation. These physicochemical properties are critical, as rectal release of active constituents at body temperature depends on such characteristics (Hua, 2019). An increase in suppository hardness was observed after 2 months, which may be attributed to gradual moisture loss from the formulation matrix. Despite this change, hardness values remained within an acceptable range for patient administration. Furthermore, *in vitro* release profiles remained largely consistent over the 2-month period, with only a slight reduction in activity.

While the present study highlights the promising anti-inflammatory activity of *P. pellucida* using commonly employed *in vitro* assays, several limitations should be acknowledged. Reliance on *in vitro* methods provides only preliminary evidence and does not account for the complex biological processes that occur *in vivo*. Therefore, future studies should evaluate the drug efficacy using *in vivo* inflammation models (such as carrageenan-induced rat paw edema model) and investigate the pharmacokinetic and pharmacodynamic profiles of the formulated suppository. To enhance mechanistic understanding, future studies could incorporate differential scanning calorimetry (DSC) that assesses the thermal behavior. Mucin interaction assays and additional adhesion-related evaluations (such as texture analysis, contact angle measurement, and detachment force testing) may also be employed to better simulate suppository-rectal mucosa interactions. Furthermore, progression toward *in silico* evaluations and nanotechnology-based delivery systems (such as polymeric nanoparticles) may improve the stability and targeted delivery of bioactive constituents.

In conclusion, this study demonstrates that the aqueous extract of *P. pellucida* (L.) Kunth exhibits superior dose-dependent biological activity compared with the methanolic extract, as evidenced by multiple *in vitro* assays. Phytochemical characterization revealed a predominance of fatty acids, which may contribute to the observed membrane-stabilizing effects and inhibition of protein denaturation. Importantly, the findings extend beyond extract-level evaluation by demonstrating successful development of a herbal suppository. The optimized glycerin/PEG 4000/water-based formulation exhibited acceptable physicochemical characteristics and stability throughout the evaluated period, while the results of biological membrane permeation model further supports its functional performance.

## Data Availability

The original contributions presented in the study are included in the article/supplementary material, further inquiries can be directed to the corresponding author.
